# Refugee Women’s Receptiveness for Virtual Engagement on Reproductive Health During the COVID-19 Pandemic

**DOI:** 10.1007/s12529-022-10097-3

**Published:** 2022-05-12

**Authors:** Heike Thiel de Bocanegra, Zahra Goliaei, Nossin Khan, Sereen Banna, Rawnaq Behnam, Sheila K Mody

**Affiliations:** 1 Department of Obstetrics and Gynecology, School of Medicine, University of California, UCIMC – Chapman Pavilion, 3rd Floor, suite 3400, Irvine, Orange, CA 92868 USA; 2 Public Health, School of Social Sciences, University of California, Merced, Merced USA; 3 California Department of Health, Office of Refugee Health, Sacramento, Sacramento USA; 4 Department of Obstetrics, Gynecology and Reproductive Sciences, School of Medicine, University of California, San Diego, San Diego, USA; 5 Department of Obstetrics and Gynecology, University of California, Irvine, Orange, USA

**Keywords:** Qualitative study, Telehealth, COVID-19, Reproductive health, Refugee women

## Abstract

**Background:**

Refugee women who leave their country due to persecution and violence have multiple barriers to sexual and reproductive health (SRH) services. The COVID-19 pandemic added an additional barrier to in-person reproductive health education, dialogue, and clinical care. This study aimed to assess the potential of using virtual group meetings as a forum for refugee women to learn about and discuss reproductive health concerns such as cervical cancer screening, family planning, childbirth, and postpartum care.

**Method:**

We conducted semi-structured interviews with 36 refugee women and stakeholders to assess factors that impact refugee women’s receptiveness for virtual platforms to obtain information and engage in discussions on reproductive health. Thematic analysis was conducted using the software Dedoose.

**Results:**

Openness to engage in virtual platforms varied by refugee community, women’s demographic, and life experience. The women’s involvement with local refugee groups facilitated their engagement with virtual platforms. Furthermore, individuals’ family structure and marital relationship, along with literacy and English proficiency, and access to and familiarity with technology impacted engagement. Virtual groups needed to mirror confidentiality and women expressed a strong preference that groups were all-women.

**Conclusion:**

Refugee women are receptive to virtual groups on SRH when they are offered in a culturally appropriate manner that considers the living situations and access to technology after arrival to the USA. Findings from this study provide a framework to develop and tailor effective virtual or hybrid virtual-in-person programs for women in refugee communities.

## Introduction

Since 2008, the USA resettled 695,699 refugees and asylum seekers and granted 161,087 Special Immigrant Visas (SIV) to Afghan and Iraqi nationals and their families [1, 2]. California is one of the three top receiving states of new arrivals to the USA [3]. From 2019 to 2021, California received 4014 refugees, of whom 737 (18 percent) were from Afghanistan, and 753 (19 percent) from Iraq, Syria, Somalia, and the Democratic Republic of the Congo [3]. Forced migration is a known risk factor decreasing women’s access and utilization of reproductive health care [4]. Both pre-resettlement situations such as the different prevalence of the disease, migration trajectories, and living conditions and post-resettlement situations such as language and cultural differences, and fear and distrust of a new health care system can reduce access to appropriate reproductive health care services for newcomers [5]. After resettlement, refugee women have a higher risk of unintended pregnancy, induced abortion, and obstetric complications compared to the women of the host countries [6, 7]. For refugee women, barriers relating to language, acculturation, a gender-norm culture, or religious beliefs may decrease the ability or willingness to seek care in their new home country [8]. Prior literature indicates that forced-migrated women have limited knowledge of sexual reproductive health (SRH) and may not be aware of the many resources available in the new health care system [6]. Therefore, there is a continuous need to improve knowledge on reproductive health, available resources, and access to health care after resettlement among refugee women [9].

Refugee women reported difficulties participating in the existing interventions that support women’s SRH and less willingness to discuss reproductive health topics with clinicians in a new country [6, 10]. Prior studies reported specific methods that facilitated refugee engagement in SRH. These methods included social support, trust, provision of appropriate services, and provider’s support, which reported effective tackling of reproductive health barriers for refugees [11]. One method to facilitate refugee engagement in SRH was the use of peer support groups with refugee women meeting, discussing, and communicating their topics of concern within the group, allowing them to strengthen ties with members of their cultural community [12, 13]. These peer support groups improved the refugee women’s wellbeing and health status, increased their sense of belonging and wellbeing, and improved access to health education [14]. Group models are reported as a successful method for prenatal and postpartum care in promoting refugee women’s health. Somali refugee women reported that group prenatal care helped them feel empowered and confident, with the greatest benefits coming from storytelling with peers [15, 16]. Additionally, what the women learned from the peer support training program empowered them to acquire further learning about other issues which would become valuable for their health and wellbeing [17].

In March 2020, the World Health Organization declared the COVID-19 pandemic, which initiated a radical change all around the world. Most countries, including the USA, ordered a lockdown and used social distancing to reduce asymptomatic transmission. This lockdown impacted many sectors including health care and community health promotion. Non-essential clinic appointments were canceled or rescheduled which impacted many preventive women’s health services. Refugees like other communities of color and low-income families were disproportionately experiencing the impact of COVID-19 [18]. The rapid implementation of social distancing practices impeded the expansion of in-person cultural orientation programs as a tool for refugee women to access reproductive health information. However, it was not known whether refugees would be open to using virtual platforms such as Zoom, webinars, or groups in social media for reproductive health education, discussion, and support on reproductive health promotion and disease prevention.

This study is based on patient-centered outcome research (PCOR) principles of patient engagement in research and interventions [19–21]. We conducted a qualitative interview study, to explore refugee women’s familiarity and comfort with digital platforms including virtual group meetings and receptiveness for using these platforms for virtual engagement on reproductive health issues. Virtual engagement is defined as participation in health information groups or webinars and participation in a dialogue about barriers to reproductive health care and disease prevention. We aimed to identify elements that need to be considered when planning virtual group meetings for future refugee arrivals.

## Methodology

The California Refugee Reproductive Health Network (ReproNet) was founded in 2019 to strengthen the regional refugee-academic community partnerships and engage in dialogue with refugee communities to investigate their preferences for sexual reproductive health and well-woman care [22]. The network includes stakeholders, identified as community leaders and health and social service providers who serve refugee communities and refugee women from refugee communities in Sacramento and San Diego, USA.

ReproNet activities are based on a participatory, community-engaged approach wherein the refugee women choose a reproductive health topic of interest [23] and are given the opportunity to discuss and exchange questions, challenges, and experiences with the particular reproductive topic with a clinician or public health facilitator. These peer group meetings help establish rapport and build trusting and respectful relationships with the women.

In coordination with ReproNet stakeholders, a ReproNet research workgroup consisting of experienced researchers in qualitative interview methodology and bilingual, bicultural student volunteers developed an outline for a semi-structured interview and reviewed it with the 12-member ReproNet steering committee for appropriateness and completeness of interview prompts (see Table 1). The interview guidelines included open-ended questions to let participants talk openly around their own experiences regarding comfort with using virtual environments, the impacts of cultural and linguistic barriers on social media and virtual meetings use, and possible concerns in the community to use virtual communications for SRH topics. All members of the research team were scholars in refugee health or refugees themselves; senior members of the team had qualitative research experience and were public health researchers (HTB, ZG) and clinician scientists (SM). They provided training on the study aims and review of the interview outline and prompts. Interviews were conducted by bilingual, bicultural interviewers with experience in open-ended interviewing techniques. During the interviews, interviewers conducted follow-up questions to probe for more details or clarify a response. The follow-up prompts utilized by interviewers depended on the interviewee’s responses to previous questions.

**Table 1:** Demographic description of stakeholders (*n* = 9) and refugee women (*n* = 27)

	Stakeholders (*n* = 9)	Refugee women (*n* = 27)
Country of origin		
Afghanistan—Dari speaking	2	6
Afghanistan—Pashto speaking	1	5
Democratic Republic of the Congo	0	3
Egypt	1	0
Iraq	2	4
Somalia	1	4
Syria	2	5
Age group		
< 30 years old	1	9
30–39 years old	1	10
40–49 years old	5	6
50 + years old	1	3
Length of time in the USA		
< 5 years	2	21
6–10 years	4	1
11–19 years	0	2
20 + years	2	4

We conducted semi-structured interviews from July 2020 to September 2020 via videoconference with refugee women and ReproNet stakeholders. Stakeholders were identified through purposeful sampling among community leaders and health and social service providers who serve refugee communities in California’s study targeted areas, Sacramento and San Diego, USA.

Refugee women were from refugee communities with large number of arrivals to California in the past 10 years: Afghanistan (Dari or Pashto speaking), Iraq, Syria, Somalia, and the Democratic Republic of the Congo [3, 24]. Participants were recruited via purposive sampling with the help of the ReproNet providers and stakeholders. Purposive sampling allowed us to have an appropriate and relevant sample to capture a wide range of perspectives based on the study research question [25]. We recruited 3–6 women with diverse ages (women between 20 and 50 years) from different countries of origin to have a variety of possible positive and negative experiences with virtual settings. To maximize the quality of our sampling, we considered stakeholders who had more experience with virtual communication with refugees, and their experiences could be comparable with similar refugee community settings.

After verbal informed consent, women participated in a 30–45-min video or phone interview. The interviewers were trained to conduct qualitative interviews on reproductive health topics. If possible, the interviews were done in the interviewee’s primary language. If the interviewer did not speak the language, the questions were asked in English and interpreted by a bilingual member of the research team. If women preferred not to be audio recorded, a second research team member took detailed notes of the women’s responses.

Interviews were audio-recorded, and transcribed by audio to text software if an interview was conducted in English. A team member double checked the transcription and corrected errors. If interviewers were in a language other than English, a bilingual team member transcribed and translated the interview. An English-speaking team member read through the transcript to confirm completeness of the transcription. We used Dedoose software for data analysis [26]. Each interview was separately coded by two research team members and any inconsistencies were discussed with the research team. Using thematic analysis, research team members developed inductive codes related to factors that may impact women’s familiarity and comfort with virtual settings on reproductive health access to digital devices, social media use, telehealth usage, virtual group composition, and virtual group content [27, 28]. After creating the initial code list for each of the topics, two other research team members who did not participate in the interviews or in the initial coding developed higher order constructs of the themes and categories, which were then shared, discussed, and revised by the research team. The research team examined the emerging themes and assessed how they might vary by refugee group to better understand the differences in receptiveness for virtual settings and refugee women’s concerns across the refugee communities. We conducted a total of 36 interviews. The as research team analyzed the data as each transcript was ready, and two different research team members coded each interview. We were able to stop recruiting when the research team members concluded that study data is saturated and there are no new themes emerging from new interviews [25]. As the emerging themes included both positive and negative experiences, we did not need to include specific negative cases to disconfirming emerging themes. Final themes and findings were presented and discussed with the ReproNet steering committee to review the validity of interpretation [29]. This study was approved by the Institutional Review Boards of the University of California, San Diego, Irvine, and Davis.

## Results

A total of 27 refugee women and nine stakeholders participated in the study. Two-thirds of the 27 refugee women were less than 40 years old. The majority of the women arrived in the USA less than 5 years ago, but 4 women arrived over 20 years ago (see Table 2). Five of the 9 participating stakeholders were involved with community-based organizations at various levels. Other stakeholders included a range of health care clinicians who work with refugee women. Most of the stakeholder interviewees were identified as women (*n* = 8).

**Table 2:** Survey questions refugee women’s readiness for virtual engagement on reproductive health

**Theme**	**Question introducing interview segment**
**1. Familiarity with refugee community (warm-up question)**	Can you tell me how you have been involved with the [refugee] community with regards to women’s health?
**2. Refugee women’s access to technology**	What can you tell me about the refugee community’s familiarity with communication devices (i.e., cellphones, computers/ laptops, iPads) for women’s health?
**3. Use of technology by women**	How do women use the internet or cellphones to communicate about women’s health (i.e., for personal communication, text messaging, international calls)?
	Can you tell me about positive and negative experiences with the use of technology that you heard or are aware of? (Connection to family back at home, connection to local community)
**4. Impact of COVID-19 on refugee women’s health care access**	Did anything change in recent months due to COVID-19 regarding women’s health care access (i.e., zoom for lectures, telehealth, virtual programs)? How well did women adapt to these changes?
**5. Potential for use of virtual communication** **(The follow-up prompt depends on previous responses)**	What is the potential of using virtual groups for women to inform them about reproductive health issues? What is the potential of using virtual groups for women to engage women in a conversation about reproductive health issues?
**6. Additional resources**	Is there somebody else we should talk to (i.e., community leaders)? We would like to interview refugee women who can tell us more about their experiences, can you suggest how we should recruit them?

We identified several themes that impacted women’s receptiveness to engage in virtual platforms about reproductive health issues: (a) Involvement with the local refugee community may facilitate engagement in virtual groups; (b) Adoption of new technologies is hampered by language and literacy barriers; (c) Family structure and household environment impact access to the internet and social media; (d) Access and familiarity with technology facilitates the use social media and videoconferencing; (e) Prior experiences with informal virtual groups impact interest in virtual groups; (f) Confidentiality and all-women groups are important features for virtual group meetings; (g) Refugee women are reluctant to use telehealth services.

### Themes

#### Involvement with the Local Refugee Community May Facilitate Engagement in Virtual Groups

Across all interviewed refugee groups, some women reported feeling isolated upon arrival due to the absence of friends and family in their new areas. They also reported feelings of anxiety and isolation following their arrival. Women found that family-centeredness and isolation are typical to their community as described in the following comment:

*Afghan women primarily are here by themselves. They don*’*t have any other family members besides their children and husbands. So, they*’*re mostly focused on whole household tasks like preparing dinner, taking care of the children. [Pashto speaking Afghan refugee, mid 30s]*

Nevertheless, some women were able to integrate into their local refugee communities or create new social networks, for example, by being part of social media groups.

*I feel very much integrated very much connected to my community here … the neighborhood that I*’*m living in here is all Afghans and I am very much in contact with them. [Dari speaking Afghan refugee, early 30s]*

Although community leaders and social networks have the potential to facilitate interest and participation in virtual groups, participants in this study suggested that outreach to the refugee community needs to consider that some women might also be isolated from their local refugee community.

Existing connections among community members and with community-based organizations can play a crucial role in disseminating information about communication resources including support groups. As an example, a Syrian woman created a group on Facebook that facilitates community outreach to build connections among community members and leaders. However, some women were reluctant to be involved with community-based organizations or certain CBO staff out of distrust or concern about being used for the organization’s fundraising or publicity.

*I don’t think it’s a good idea to contact [organization] because nobody likes [organization] …nobody will want to be in a group organized by [organization]. [Arabic speaking, Syrian refugee, 27 years old]*

#### Adoption of New Technologies Is Hampered by Language and Literacy Barriers

Some of the participants noted that women generally discontinue their education once they get married, occurring often at a young age. As a result, many women in those communities only have a high school level of education or less. In both Syrian and Afghan participants, education and use of technology differed by the women’s province and territory of origin and might to limited to basic technology lessons. Arab countries, and regions within these countries, also vary in women’s access to education and technology use based on how “developed” and conservative countries are. One of the interviewees who worked with Afghan parents in a school commented:

*Everything is in English so a person that cannot know the letters in English so how they search something or how they turn zoom on. [Pashto speaking Afghan refugee, early 30s]*

Education encompasses literacy in the native language and is associated with the ability to become proficient in English after arrival. Some participants reported that women with higher education levels may have used technology in their country of origin and can adapt to technology more easily. When women had attended college, community programs, or worked outside of their home, they were more likely to be familiar with Zoom and other technology. Virtual technology is harder for women who cannot read and write in their native language and it compounds their difficulty to learn. In addition, some participants reported that limited English proficiency impedes their use of technology. The familiarity of technology also differed by age. Younger women are more likely to be familiar with social media use than older women.

*I was a teacher back in Afghanistan for 22 years. So, I knew how to use phone back from Afghanistan. So, I don*’*t really have any problem with my phone. [Dari speaking Afghan refugee, 44 years old]*

*I use the laptop for work. I am a breastfeeding educator for the Somali community. I use the computer to enter data for the system used by my work. [Somali refugee, 50 years old]*

*The older woman that*’*s harder for them to use. It*’*s a source of frustration because they*’*re not sure how to navigate through this kind of device. [Egyptian stakeholder, 48 years old]*

#### Family Structure and Household Environment Impact Access to the Internet and Social Media

In some refugee communities, women may lack the confidence to interact with agencies outside of their home due to cultural barriers. Some Afghan and Iraqi interviewees mentioned that they usually do not attend meetings or appointments without their husbands being present. Somali women also reported that refugee women primarily stay at home and take care of the children, but they did not comment on the need to involve their husbands in interactions outside of the home.

*The big barrier, I*’*m seeing for women is the lack of education that they have and the lack of English language that they have and because they are called self-confidence is very low, they couldn*’*t like can find the courage to go shop with themselves, to go to the school to see the progress of the child, to go to the doctor. [Pashto speaking Afghan refugee, early 30s]*

More so, we identified that in some refugee communities there is a strong dependence on partners’ approval and support for interactions outside of the home. This dependence was recognized to extend to women’s comfort to use social media within the Afghan community. In some Afghan families, women either prefer or need their husband’s approval to use social media or to share something in a group setting. Potential stigma and loss of reputation to be a respectable woman might also impact women’s comfort in using social media. This can be attributed to the ability of social media to expose people’s private lives and publicize personal information to individuals who are outside the family. One Afghan interviewee expressed women’s dependence on their spouses as a cultural practice:

*Women don’t have absolute permission to do everything by their own, they always need permission from their husbands or their families. Like whenever they want to share something in a group or whenever like they want to provide some information or get information from others. And yeah, it depends on how their families were a little react on these things. [Dari speaking Afghan refugee, early 30s]*

Some Iraqi women commented on reluctance to connect with other women via social media and virtual group meetings but added that there are variations by cultural background and women’s personality.

*I feel like it depends on the woman. It depends on where they’re coming from. Every country differs, especially Arab countries, given that we’re conservative. But there is development, and there are countries where it is normal for people to listen to women and for women not to be concerned about what men have to say. [Arabic speaking Iraqi refugee, 21 years old]*

*Of all the women I know I am the only coward. I am the only one who is hesitant because I need to ask my husband and I need to know what he is okay with and is comfortable with when I am taking part in it. [Arabic speaking Iraqi refugee, 43 years old]*

#### Access and Familiarity with Technology Facilitates the Use Social Media and Videoconferencing

With regard to technology access, most of the women have smartphones and at least one computer per household. However, the devices being used are sometimes basic and internet access is limited. In terms of usage, they have limited ability to use the applications that come with the devices.

*Mostly they*’*re using the cell phones that they get for free which don’t have SIM cards. They*’*re just whenever they*’*re at home, they*’*re using their home Wi-Fi for communication. [Dari speaking Afghan refugee, early 30s]*

*Internet can be slow, sometimes it gets stuck our internet is disconnected. Women may not know how to connect back the internet system in the house. [Pashto speaking Afghan refugee, early 30s]*

Most women reported moderate to advanced ability in using Facebook and sometimes reported watching educational videos on YouTube. However, their familiarity with conducting Google searches was limited, especially searches in English. Across the refugee groups, the main differences by demographic groups in the use of computers, Zoom, and other applications are by education and associated familiarity with the use of technology in the home country, age, and ability to have training.

Education is associated with literacy in the native language and the ability to become proficient in English after arrival. Some participants reported that women with higher education levels may have used technology in their country of origin and can adapt to technology more easily. However, computer instruction in the home country was very basic, if it happened at all.

*The only [lessons] was [to learn] how to open and close the laptop. It wasn’t that advanced in our schools to teach us basic skills and how to use computer. It was mostly on paper and pen. And you know that until a girl gets to seventh grade they start getting married. But now people are advancing, and they do not get married that early." [Arabic speaking Syrian refugee, 27 years old*]

I was a teacher back in Afghanistan for 22 years. So, I knew how to use phone back from Afghanistan. So, I don’t really have any problem with my phone. [Dari speaking Afghan refugee, 44 years old]

The familiarity of technology also differs by age. Younger women are more likely to be familiar with social media use than older women.

*The older woman that*’*s harder for them to use. It*’*s a source of frustration because they*’*re not sure how to navigate through this kind of device. [Egyptian stakeholder, 48 years old]*

The participants in this study mentioned an interest in completing trainings or courses to assist in their use of technology. Some of the women relied on family members (older children, husband) to help with accessing social media and troubleshooting technological issues. Conversely, some women with school-age children, who use Zoom for virtual classes, received guidance on how to use Zoom. While this experience may help women to attend virtual groups for themselves, supervising the virtual classes of their children might also create additional stress for the women.

*I didn*’*t know what Zoom is or how to use it, but my children who are in school have taught me. This is my son who is young but often times teaches me what to do. Of course, the other women experience the same thing wherein their children guide them. [Arabic speaking Iraqi refugee, 43 years old]*

*There are some stuff that are hard for me, so I have to ask my kids. Of course, if there was an interpreter tell me “press here, press there” I would learn. Because now I depend on my kids I just give them the phone and they log me in and they give it to me ready. [Arabic speaking Syrian refugee, 33 years old]*

In addition to access, privacy in the home was at times a challenge. The participants from Afghanistan reported families to tend to be large and in crowded housing conditions; 5–10 children and tend to live in one- or two-bedroom apartments. This limits the women’s capability of attending Zoom meetings that require private settings.

#### Prior Experiences with Informal Virtual Groups Impact Interest in Virtual Groups

Women are familiar with phone applications for personal uses, including the use of WhatsApp and Viber for international calls to family and friends abroad. The use of computers, Zoom, and other applications and use of devices for group chats varies, but women often utilize chat groups for non-health purposes (to sell items, share news, etc.). These groups serve many functions, including posing health-related questions and information. In some instances, chat members with medical training provide advice or use the group to share health information in the group’s spoken language. Women with health concerns might seek health care advice from such forums rather than from a doctor; nonetheless, concern about the correctness of this advice due to the insufficient monitoring of reputable sources was expressed by the resource persons.

*I have a group with doctor friends on WhatsApp and some of them are specialists and train us. Sometimes we joke on the group and other times we share pertinent information about COVID and share resources. Sometimes I get information from doctors that are important that I like to share. Sometimes you get false information. [Dari speaking Afghan stakeholder]*

Women were asked to provide suggestions for successful virtual groups. Most of these suggestions were provided by the stakeholders who at times had experience in organizing groups within their professions. Some suggestions indicated that to participate in virtual groups women needed to get buy-in from their male partner or have their husbands meet with the group facilitator for clarification on and approval of the subjects being discussed.

*I would recommend to have maybe the first time we had a meeting with them have their husband and wife be together on camera to explain everything. I think that would make them a lot more comfortable. Don*’*t even ask them. Say, well, we*’*d like to have, you know, for you to be involved. We like to talk to you and your husband. [Pashto speaking stakeholder]*

In addition, several participants commented that incentives in recognition of the time for participating and engaging in virtual groups might increase attendance as well as increase the value of the meetings for their male partners. Another motivating factor for participation and engagement was providing women with the ability to choose the topic for the virtual groups, perhaps from a list of topics, so that the meetings address their interests and needs. Specifically, some recommended starting a series of virtual group meetings with less sensitive topics before talking about reproductive health. Some participants also requested videos and visuals in their native language to watch and some also preferred having written materials in advance to study prior to the virtual group.

*But in regards to reproductive health, definitely, you know, painful things that they*’*re experiencing or being in different parts of their, you know, concerning their reproductive health and bodies, so pain, birth control, you know, maybe that kind of issues. You*’*ll have to ease into it, you can’t just jump into it. [Egyptian stakeholder]*

#### Confidentiality and All-Women Groups Are Important Features for Virtual Group Meetings

Among the many interviewees, several of the participants indicated that knowing each other and having the option to ask questions to a facilitator was vital for their participation in a virtual group.

*It totally depends on who*’*s going to be in the chat room and what issues are going to be discussed and the level of comfort and trust that is established between the people who are going to be. [Egyptian stakeholder]*

*If, like if they know the rest of the women inside the group, they might be like more conservative and sharing like their opinions or sharing their experiences because like you, she knows me, she might like say something to someone else that we know or she might share this information. [Iraqi stakeholder]*

Concerning confidentiality, women felt strongly that the virtual groups should be limited to women, facilitators, and interpreters. One participant suggested asking all women to have the camera on at the beginning of the meeting to ensure that there are no men present. Some participants, conversely, requested keeping their camera off and using nicknames during the group meetings. Women might also be more engaged if they have the option to pose questions privately to the moderator during the group chat.

*It’s critical it’s just a group of women. There shouldn’t be a man in the group, not even an interpreter. They think these issues are so private that they don’t feel comfortable sharing this information with a man. [Dari speaking Afghan stakeholder]*

*I think everyone must open a Camera if it’s through zoom so everyone makes sure that everyone is female attending the zoom call. We make sure that there’s no man in the zoom meeting. [Arabic speaking Syrian refugee, 20 years old]*

To facilitate privacy, several participants recommended scheduling the virtual group meetings during the times women are home alone. Since husbands and children are home all days and some families are confined to small apartments due to social distancing rules, this privacy became difficult during the COVID-19 pandemic.

#### Refugee Women Are Reluctant to Use Telehealth Services

Due to the risk of COVID-19 infection, most clinics canceled in-person appointments and rescheduled video appointments, when clinically appropriate. Several of the participants reported a lack of familiarity with utilizing telehealth. There was a strong preference for in-person doctor visits. *So, it’s super hard for me because they created a program called My Chart so we can follow up with his [the doctor’s] appointments …we will wait for the appointment or try to open the appointment at the right time and then we would lose connection and then we try to reconnect again. [Arabic speaking Syrian refugee, 27 years old].*

*[Seeing a doctor] face-to-face is still easier. It’s still hard for people, old people, who don’t know how to use a phone. I didn’t know how to use a laptop. [Arabic speaking Syrian stakeholder, late 30s]*

*Especially if it’s a doctor like you want to sit with him and understand what’s wrong from him. For example, like he asks you over the phone and it’s very fast. And the whole meeting takes only five minutes…And you even forget most of the topics you want to talk about questions you want to talk about. [Arabic speaking Syrian refugee, 33 years old]*

While in the past clinics would help patients to schedule in-person screening appointments, such as for Pap smear and mammograms, some participants reported that these calls became scarcer since the COVID-19 pandemic and lack of information hampered their abilities to seek out telehealth alternatives. Some of the participants also reported seeking care from emergency rooms due to the decrease in in-person clinic access.

*“…some of them are not like quite convinced with it as an alternative to an actual clinic visit and that*’*s why you see our ER visits have gone way much higher in the past months than before, because like they go to the ER and say: ‘ Well, I did a telehealth appointment visit and he told me that I have that and that and then they prescribed a medication, or they send me for a test.’ ” [Iraqi stakeholder]*

Women also reported issues with hearing and understanding the doctor, interruptions callers, and internet problems. A few participants reported a, possibly cultural, fear of being on camera. One interviewee, a 50-year-old Somali refugee, indicated that women have not tried to communicate via video communication with one another because of privacy concerns and having to cover up in hot weather. Several participants stated that the younger members of the community might navigate telehealth easier than the older ones.

## Discussion

The sudden need for virtual platforms due to the COVID-19 pandemic challenged both clinicians and communities to explore new ways to continue community health education. Virtual interactions such as telehealth visits, social media, and virtual group meetings became the priority focus of attention and growth to engage women in health promotion and disease prevention [18, 22]. In our study, we showed the potential and challenges of virtual meetings on reproductive health among refugee women coming from five different countries.

The diversity of refugee participants with regard to native country, health literacy, and age in this study illustrates the variance in comfort with adopting and using technology and social media for reproductive health. In general, local refugee communities that are built on strong personal connections can facilitate comfort using virtual groups even about sensitive issues [30]. Most women in this study had access to and basic familiarity with technology. However, their level of education and age impacted their utilization of social media and virtual platforms for information sharing and group conversations. Comfort with sharing information online varies by country, region, or linguistic background and prior positive or negative experiences in using virtual platforms.

This study is novel since it is one of the first studies to explore the utilization of virtual platforms among refugee women living in the USA and community stakeholders. Since the study included very practical questions regarding access to technology and receptiveness to virtual patient educational groups, this information could be rapidly utilized to help address technology barriers. The participants from this study ranged from women who were familiar and comfortable using technology to women who needed family or program manager support to enter the virtual interview. The participants also reported challenges and reluctance of using telehealth services. This reluctance to use telehealth services among low-income and minority communities has also been observed in other studies [31, 32]. This reluctance may result in delayed preventive health care for refugee women and their families.

There are several limitations of the study. Some of the interviews relied on an interpreter; therefore, participants may not have elaborated some of their responses. The interviews were done via telephone or videoconference while the women were at home and homeschooling children; hence, their responses may have been impacted by the presence of family members close by. In addition, given the interviews were done via phone or computer and not done in person, this study did not capture the virtual platform receptiveness of refugee women without any access to phone or computer. Lastly, although the study included purposefully diverse sampling, there were only a few women interviewed from each refugee group. Therefore, the results of the study cannot be overgeneralized.

The future implications of this study’s findings include informing a refugee readiness assessment checklist that can guide future transitions to virtual or hybrid virtual-in-person programs for refugee communities [22]. This checklist can be used by community organizations hoping to utilize virtual platforms among refugee communities and can be used to systematically assess the experiences, resources, and limitations of using technologies with refugee groups and to elicit details that contribute to culturally appropriate planning and implementation of refugee virtual group meetings. This toolkit can enable program planners and community leaders to efficiently identify strengths and gaps in refugee communities and initiate a dialogue with the community. Future studies could evaluate specific interventions that address refugee women’s reproductive health issues through the improvement of access to telehealth and virtual groups and integration of technology with in-person services.
